# Distribution of Hepatitis C Risk Factors and HCV Treatment Outcomes among Central Canadian Aboriginal

**DOI:** 10.1155/2016/8987976

**Published:** 2016-04-17

**Authors:** Parmvir Parmar, Daniel J. Corsi, Curtis Cooper

**Affiliations:** ^1^Faculty of Medicine, University of Ottawa, Ottawa, ON, Canada; ^2^The Ottawa Hospital Research Institute, Ottawa, ON, Canada; ^3^The Division of Infectious Diseases, Department of Medicine, University of Ottawa, Ottawa, ON, Canada

## Abstract

*Background.* Aboriginal Canadians face many lifestyle risk factors for hepatitis C exposure.* Methods.* An analysis of Ottawa Hospital Viral Hepatitis Clinic (Ottawa, Canada) patients between January 2000 and August 2013 was performed. HCV infection risk factors and HCV treatment outcomes were assessed. Socioeconomic status markers were based on area-level indicators linked to postal codes using administrative databases.* Results.* 55 (2.8%) Aboriginal and 1923 (97.2%) non-Aboriginal patients were evaluated. Aboriginals were younger (45.6 versus 49.6 years, *p* < 0.01). The distribution of gender (63.6% versus 68.3% male), HIV coinfection (9.1% versus 8.1%), advanced fibrosis stage (29.2% versus 28.0%), and SVR (56.3% versus 58.9%) was similar between groups. Aboriginals had a higher number of HCV risk factors, (mean 4.2 versus 3.1, *p* < 0.001) with an odds ratio of 2.5 (95% confidence interval: 1.4–4.4) for having 4+ risk factors. This was not explained after adjustment for income, social deprivation, and poor housing. Aboriginal status was not related to SVR. Aboriginals interrupted therapy more often due to loss to follow-up, poor adherence, and substance abuse (25.0% versus 4.6%).* Conclusion*. Aboriginal Canadians have higher levels of HCV risk factors, even when adjusting for socioeconomic markers. Despite facing greater barriers to care, SVR rates were comparable with non-Aboriginals.

## 1. Introduction

Chronic infection with HCV can lead to liver fibrosis, hepatocellular carcinoma, end stage liver disease, and the need for liver transplant [1–4]. HCV infection is also associated with higher costs to healthcare systems worldwide [4–7]. The prevalence of HCV infection in Aboriginal Canadians is 5–8 times that of non-Aboriginal Canadians [8–10]. Furthermore, the rate of new HCV infection amongst Aboriginals is 2.5 times that of the general Canadian population [9]. Aboriginal Canadians are disproportionately infected with HCV owing to higher rates of injection drug use, cocaine use, and incarceration [9–13]. Although recent studies suggest that some Aboriginal groups (from coastal BC and SE Alaska) may be less likely to progress to chronic infection, overall HCV-related mortality remains higher in Aboriginals [14–18]. Despite higher rates of HCV infection and disease burden, Aboriginals remain underrepresented in community based treatment programs [8, 9, 13, 19]. Higher rates of type II diabetes, alcoholic liver disease, nonalcoholic fatty liver disease (NAFLD), substance abuse, mental illness, and coinfection with HIV and HBV act as barriers to receiving HCV treatment in Aboriginals [15–18, 20]. Social factors (lower SES status in some Aboriginals) also act as barriers to HCV antiviral treatment access in Aboriginals and may partially explain the underrepresentation of Aboriginals in HCV treatment programs [8, 19, 21].

Despite challenges of collecting data among Aboriginal HCV populations, preliminary studies assessing HCV treatment outcomes have suggested that Aboriginal and non-Aboriginals can achieve similar SVR rates on therapy consisting of interferon and ribavirin [8, 16, 20]. The number of studies assessing HCV treatment outcomes in Aboriginals is limited, and there are no studies assessing HCV treatment outcomes in Aboriginals on triple therapy with a protease inhibitor or on a regimen of direct acting antivirals (DAAs).

The purpose of this study was to characterize levels of risk factors among Aboriginal patients and examined the extent to which differences in the distribution of risk factors could be explained by markers of socioeconomic status. We compared HCV work-up and rates of sustained virologic response (SVR)* on interferon-based regimens* between Aboriginal and non-Aboriginal patients.

## 2. Methods 

A cohort database analysis of patients followed at The Ottawa Hospital (Ottawa, Canada) Viral Hepatitis Clinic between 2000 and August 2013 was performed. The catchment area for referrals includes Eastern Ontario, Western Quebec, and Nunavut. Patients 18 years of age or older at the time of enrollment and those chronically infected with HCV (defined as remaining HCV RNA positive for longer than six months after initial exposure to the virus) were included in this study. Those patients who were HCV seropositive but PCR negative were excluded. Demographic data (including self-identified Aboriginal status), HCV risk factors (HIV coinfection, history of mental illness, history of injection drug use, tattoos, history of cocaine use, sexually transmitted infections, alcohol use, and history of incarceration), HCV treatment regimens, HCV treatment duration, and adverse on therapy events were captured using The Ottawa Hospital Viral Hepatitis Clinic database, patient charts, and electronic medical records. Laboratory test results including HCV genotype and viral load and transaminase (AST, ALT, and GGT) levels were captured at baseline.

Information on the socioeconomic status of patients was extracted using data from the 2006 and 2011 Canadian census databases. Each patient was assigned to a census dissemination area using their recorded postal code. Based on this, area-level socioeconomic markers were attributed to each patient. The socioeconomic variables included in the analyses were median pretax income, social and material deprivation, housing suitability, and urban/rural location of residence. Social and material deprivation capture dimensions of relative disadvantage at the area level and are comprised of multiple census indicators [22]. Material deprivation is based on levels of education, employment, and income while social deprivation is related to single-parent families, people living alone, and those who are separated/widowed or divorced. Material deprivation is closely related to poverty and economic disadvantage while social deprivation captures social capital and social isolation.

A summary variable for HCV risk factors was derived by combining each of the eight HCV risk factors into a single score with each component variable receiving equal weight. The proportion of individuals with four or more risk factors was also calculated to represent “high risk.” HCV outcomes were measured by fibrosis stage (METAVIR stages F0–F4) on liver biopsy or calculated stage (F0–F4) obtained from transient elastography (Fibroscan) assessment and SVR rates. In cases where individual patients received multiple biopsies or Fibroscan tests, the results of the most recent test were included. Secondary outcomes included completion of treatment and premature interruption of therapy due to side effects, serious adverse events, mental health concerns, substance abuse issues, failed virologic response, or loss to follow-up. In cases where multiple contributing factors to patients abandoning HCV therapy were identified, only the primary reason for abandoning therapy was included.

Demographic characteristics and risk factors were analyzed descriptively and reported as frequencies, percentages, mean ± SD, or medians and interquartile range as appropriate. Baseline characteristics between the Aboriginal and non-Aboriginal groups were compared using *χ* ^2^ and *t*-tests for categorical and continuous data, respectively.

Logistic regression analysis was used to assess predictors of high levels of HCV risk and, among those who had initiated treatment, SVR. Variables included Aboriginal status, sex, age, fibrosis stage/score, HCV genotype, HCV viral load at baseline, and HIV status. Variables with *p* values of <0.10 in the univariate analysis were subsequently assessed using a multivariate logistic regression alongside well-established predictors of SVR [23, 24].

All data were analyzed using SPSS version 17.0 (SPSS Inc., Chicago, Illinois) and Stata version 12 (College Station, TX). *p* values of less than 0.05 were considered statistically significant. The Ottawa Hospital Research Ethics Board approval and informed consent from patients were obtained for the use of this data for research purposes.

## 3. Results

A total of 55 (2.8%) self-identified Aboriginal and 1923 (97.2%) non-Aboriginal HCV-infected patients residing in Eastern Ontario, Western Quebec, and Nunavut were included in the analysis. Aboriginals were younger with a mean age of 45.6 years versus 49.6 years in non-Aboriginals (*p* < 0.01). The proportion of males (63.6% versus 68.3%), HIV coinfected (9.1% versus 8.1%), genotype 1 infected (68.5% versus 65.4%), HCV viral load at baseline (3.87 × 10^6^ versus 3.95 × 10^6^ IU/mL), and advanced fibrosis stage (F3-F4) (29.2% versus 28.0%) were similar (all, *p* > 0.10) (Tables 1 and 2).

**Figure 1:**
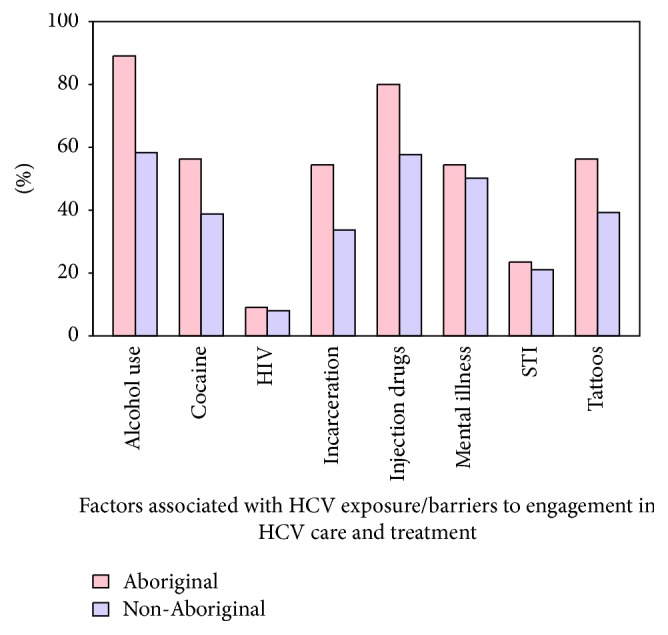
Factors associated with exposure and which act as barriers to engagement in HCV care and treatment (Aboriginal (*n* = 55) and non-Aboriginal (*n* = 1923) patients).

The prevalence of factors associated with HCV exposure and which act as barriers to engagement in HCV care and treatment are listed in Figure 1.  Aboriginals had a higher prevalence of injection drug use history, tattooing, cocaine use, alcohol use, and incarceration (*p* < 0.01). Aboriginals had a higher total number of risk factors (mean 4.2 risk factors versus 3.1, *p* < 0.0001) and a greater proportion with four or more risk factors (67.3% versus 45.1%, *p* = 0.001) compared to non-Aboriginals.

Census data revealed a trend toward higher levels of social deprivation and poor housing suitability among Aboriginals. Aboriginals came from neighborhoods with greater levels of poverty (47.2% versus 34.2%) compared to non-Aboriginals (*p* = 0.049) and with higher levels of material deprivation (31.4% of Aboriginals in most deprived quintile versus 22.4%, *p* = 0.023). Census data also indicated that 67.3% of Aboriginals and 75.0% of non-Aboriginals live in medium/large urban centers (*p* = 0.19), whereas 21.8% of Aboriginals and 14.9% of non-Aboriginals live in rural areas, respectively (*p* = 0.16).

In this cohort, Aboriginal and non-Aboriginal patients had similar rates of HCV treatment initiation (Aboriginal = 36.4% and non-Aboriginals = 40.9% (*p* = 0.50)) (Table 2). The rate of multiple (>2) rounds of HCV therapy was similar between Aboriginals and non-Aboriginals (5.5% versus 6.1%, *p* = 0.85). All Aboriginal patients on therapy received interferon-based treatments and no Aboriginal patient initiated a treatment regimen containing a direct acting antivirals (DAAs). In comparison, 4.7% of non-Aboriginal patients initiated DAA-containing regimens with or without interferon (*p* = 0.10). Aboriginal patients interrupted therapy more often than non-Aboriginals with 40.0% completing treatment compared to 60.9% in non-Aboriginals (*p* = 0.06). Aboriginals were more likely to discontinue treatment due to loss to follow-up, adherence concerns, and/or substance abuse issues (25.0% versus 4.6% in non-Aboriginals, *p* < 0.0001). Despite these challenges, SVR rates were similar by group (56.3% versus 58.9%, *p* > 0.10).

Being of Aboriginal identity was associated with an age adjusted odds ratio of 2.19 (95% confidence interval [CI]: 1.22–3.91) for having four or more HCV risk factors/barriers to engagement in care exposure risk factors compared to non-Aboriginals (Table 3). Other characteristics associated with increased odds of having clustering of HCV risk factors included being male, having HCV genotype 1 infection, and being from neighborhoods with high social or material deprivation and low levels of income (*p* < 0.01). Receiving blood transfusions was associated with lower odds of having cluster HCV risk. In a mutually adjusted model which accounted for characteristics of HCV infection and markers of socioeconomic status, being of Aboriginal identity remained a strong predictor of having clustered HCV risk with no attenuation in the odds ratio (OR 2.36, 95% CI: 1.22–4.59).

The associations between Aboriginal status, established predictors of SVR (age, HCV genotype, HCV viral load, hepatic fibrosis), and clustering of HCV risk factors were analyzed in age and sex adjusted models and a final mutually adjusted model (Table 4). The age and sex adjusted and mutually adjusted models suggested that Aboriginal status in itself does not independently predict the likelihood of achieving SVR (age and sex adjusted OR 0.78, 95% CI: 0.28–2.16; mutually adjusted OR 0.79, 95% CI: 0.26–2.40). In the mutually adjusted model, residing in low income neighborhoods was associated with a lower odds of achieving SVR (OR 0.56, 95% CI: 0.37–0.84 for viral load and OR 0.65, 95% CI: 0.44–0.96 for low income neighborhood). Having multiple HCV risk factors was not associated with a reduced likelihood of achieving SVR (OR 0.94, 95% CI: 0.67–1.34 for clustered HCV risk).

## 4. Discussion

Although based on cohort followed primarily in the predirect acting antiviral era, the results of this analysis are revealing. First, Aboriginals in this population had a greater proportion of HCV risk factors and barriers to engagement in care compared to non-Aboriginals. This underscores the need to develop complex strategies to address HCV infection in the Aboriginal population due to multiple concurrent risk factors for exposure and difficulties in accessing care and treatment. Second, Aboriginals in our population were younger but had similar or slightly higher levels of fibroses and rates of progression which may in part be related to concurrent alcohol use in the population. Third, our findings demonstrated that HCV workup rates and SVR rates were similar between Aboriginals and non-Aboriginals indicating comparable treatment outcomes among those Aboriginals and non-Aboriginals that are able to access HCV care. Finally, our socioeconomic analysis indicates that the Aboriginal population in this clinic was more likely to be resident in a low income area with greater levels of material and social deprivation. In addition, the association between Aboriginal identity and HCV risk factors/barriers to care and treatment persisted after adjustment for markers of socioeconomic status suggesting that other factors such as psychosocial factors or social isolation may be driving the increased level of risk factors among the Aboriginal population.

Previous studies have suggested that the distribution of HCV risk factors among Aboriginals and non-Aboriginals were similar [10, 12, 16]. We found clear evidence of an increased burden of risk factors among Aboriginals with more than two-thirds of the Aboriginal patients in this cohort had a clustering of four or more risk factors compared to less than half of non-Aboriginals. The literature has also suggested that HIV coinfection is higher among Aboriginals in certain populations. We found similar rates of HIV between Aboriginals and non-Aboriginals in this cohort [9, 16, 25], with the differences in risk factors mainly due to higher rates of substance abuse (alcohol, injection drug use, and cocaine use), incarceration, and tattooing among the Aboriginal population. This HIV prevalence finding may reflect an era effect and the specific characteristics of our Eastern Ontario-based Aboriginal population. High HIV incidence and prevalence rates in Central and Western Canadian Aboriginal populations represent a current critical issue requiring urgent attention [16, 21, 26].

We found that Aboriginal and non-Aboriginal patients presented to clinic with similar levels of hepatic fibrosis, despite the Aboriginal patient's being on average 4 years younger than non-Aboriginals. This finding suggests that either our Aboriginal patients were chronically infected with HCV at an earlier age or that the rate of disease progression (hepatic fibrosis) is more rapid in Aboriginals. Studies evaluating factors associated with accelerated HCV disease progression have identified alcohol use; smoking; coinfection with HBV, HIV, and schistosomiasis; insulin resistance; and diabetes as key predictors [3, 27]. A high body mass index (BMI) might also be associated with more rapid disease progression [3]. The metabolic syndrome associated risk factors are of particular importance in studying Aboriginal cohorts as they are known to be at higher risk of developing obesity and diabetes than the general population [3, 17, 18]. We found higher rates of alcohol and substance abuse in our Aboriginal cohort, but it is unclear if this is sufficient to explain our findings. Although we screened for causes of nonviral hepatitis including nonalcoholic fatty liver disease (NAFLD) and nonalcoholic steatohepatitis (NASH) at intake, we were unable to perform* a posteriori* subgroup analysis with respect to metabolic syndrome associated factors (BMI, diabetes, and/or insulin resistance) as our database did not capture these variables.

Consistent with our findings, two previous Canadian studies have indicated that HCV treatment outcomes were similar between Aboriginal and non-Aboriginal patients [8, 20]. Aboriginal patients treated with pegylated interferon 2a and ribavirin did not reveal any differences in the number of serious adverse events or reasons for discontinuation of therapy between Aboriginals and non-Aboriginals [8, 20]. Our findings indicated that Aboriginals were more likely to discontinue interferon-based therapy due to loss to follow-up/substance abuse issues compared to non-Aboriginal patients. Although SVR rates are similar between Aboriginals and non-Aboriginal groups, barriers to HCV treatment remain substantial for Aboriginals [8, 16, 20]. Aboriginals continue to be underrepresented in community treatment programs in Canada. The population assessed in this analysis was censored just at the beginning of the DAA era. Our data suggest that Aboriginal patients have yet to benefit from these newer HCV treatments including triple therapy with a protease inhibitor or combination DAA regimens, likely due to barriers acting to impede uptake in this group and cost. Given the improved dosing and safety characteristics of interferon-free combination DAA regimens, we believe that major inroads into expanding care in the Aboriginal population and other marginalized groups are feasible and should be explored.

It has been suggested that poor socioeconomic conditions, substance abuse, and mental illness are barriers preventing Aboriginal patients from initiating HCV therapy [8, 19, 21]. Access to high quality HCV care and treatment may be limited in reserve or remote areas with greater Aboriginal populations. Our findings indicated that maternal and social deprivation, low income, and poor housing conditions were not responsible for the increased levels of HCV risk factors among Aboriginal patients. This suggests that other pathways which we have not captured (e.g., social or economic isolation or psychosocial dysfunction) may be important drivers for the high levels of HCV risk factors among Aboriginal populations in Canada. Recent work suggests that underlying trauma (childhood sexual abuse, unplanned pregnancy, and personal or familial involvement with the residential schooling system in Canada) experienced by young Aboriginals influences certain high risk behaviors that predispose this group to infection with HCV and that this group is unique and may actually represent a “high risk group within a high risk group” with respect to HCV risk factors [11, 16]. Our database does not capture past traumatic events/characteristics that may underlie the development of high risk behaviors for HCV exposure but represents an important focus for future research.

Several limitations related to this analysis are acknowledged. First, the study was based on an HCV-infected population which could not be used to derive prevalence among Aboriginal and non-Aboriginal groups. It has been suggested that the prevalence of HCV is as much as seven times greater among Aboriginals compared to non-Aboriginals in Canada. Second, there were relatively few Aboriginal patients in this population (about 3% of this cohort) which may reflect difficulties of engaging Aboriginal populations in an urban-based tertiary care clinic. The small number of Aboriginals may have limited the statistical power of some comparisons. Specifically, low numbers of genotype 3 infected Aboriginals precluded evaluation of risk factor clustering. Therefore, true differences between Aboriginals and non-Aboriginals may be underestimated or perhaps over estimated due to sampling variation.

Aboriginals represent many distinct ethnic-cultural groups, and by analyzing them as one group, as we did in this study, we do not capture this diversity. However, our small number of Aboriginal patients was not conducive to perform subgroup analysis within specific Aboriginal groups (First Nations, Inuit, and Métis). Recent work shows that the Aboriginal subgroups may differ in their immune response/their ability to spontaneously clear virus [14–16, 20, 28]. Perhaps then, these differences between the various Aboriginal groups could extend into differing levels of disease burden with HCV and SVR rates. However, more studies including greater numbers of Aboriginal patients (representing many distinct Aboriginal groups) will be required to clarify this.

## Figures and Tables

**Table 1:** Baseline characteristics of HCV infection according to Aboriginal status.

	Aboriginal (*n* = 55)		Non-Aboriginal (*n* = 1923)		Total (*n* = 1978)		*p* value
	Mean/%	SD	Mean/%	SD	Mean/%	SD	
*Continuous* variables							
Age	45.6	9.4	49.6	11.2	49.5	11.1	0.010
Weight (kg)	80.1	16.4	78.1	17.7	78.1	17.6	0.437
HCV RNA (IU/mL)	3.87*E* + 06	1.02*E* + 07	3.95*E* + 06	1.37*E* + 07	3.95*E* + 06	1.36*E* + 07	0.967
ALT (IU/L)	97.3	83.7	93.6	99.6	93.7	99.2	0.786
AST (IU/L)	79.1	101.0	67.6	63.7	67.9	65.0	0.206
Fibroscan score	2.4	1.3	1.8	1.3	1.9	1.3	0.227
Biopsy stage (Metavir)	2.2	1.1	2.0	1.1	2.0	1.1	0.287
Biopsy grade (Metavir)	2.0	0.7	1.9	0.7	1.9	0.7	0.563
Fibrosis progression per year	0.151	0.168	0.126	0.254	0.126	0.252	0.625
Number of HCV risk factors	4.2	1.8	3.1	2.1	3.1	2.1	<0.001
*Categorical variables*							
Women	36.4		31.7		31.9		0.466
Genotype							0.437
1	68.5		65.4		65.5		
2	7.4		8.6		8.5		
3	22.2		18.0		18.1		
4	0.0		5.6		5.5		
5	1.9		2.4		2.4		
Four or more HCV risk factors	67.3		45.1		45.7		0.001
Material deprivation (quintile)							0.023
Least deprived	5.9		25.4		24.8		
2	19.6		20.2		20.2		
3	23.5		16.6		16.8		
4	19.6		15.4		15.5		
Most deprived	31.4		22.4		22.7		
Social deprivation (quintile)							0.227
Least deprived	2.0		9.7		9.5		
2	21.6		14.5		14.7		
3	11.8		13.4		13.4		
4	15.7		19.2		19.1		
Most deprived	49.0		43.1		43.3		
Low income neighbourhood	48.1		34.1		34.5		0.032
Poor neighbourhood housing suitability	43.1		32.9		33.2		0.127
Medium/large urban population centre	67.3		75.0		74.8		0.192
Rural area	21.8		14.9		15.1		0.16

**Table 2:** Categorical demographic characteristics and indicators of HCV management, treatment, and outcomes according to Aboriginal status.

Categorical variables	Aboriginal status		*p* value
	Aboriginal (*n* = 55)	Non-Aboriginal (*n* = 1923)	
Underwent biopsy	44.4	48.9	0.517
Underwent Fibroscan	13.0	15.3	0.64
Initiated HCV antiviral therapy	37.0	40.8	0.574
Use of erythropoietin	0.0	6.4	0.054
Treatment type			0.262
Naive	63.0	60.7	
Interferon based	37.0	34.5	
DAA ± IFN	0.0	4.7	
Treatment status			0.001
Treatment failure	10.0	13.3	
Side effects/adverse event	25.0	21.3	
Adherence/substance abuse/lack of follow-up	25.0	4.6	
Completed treatment	40.0	60.9	

Achieved SVR	56.3	58.9	0.833
Achieved SVR by genotype			
G1	60.0	54.3	0.720
G2	0.0	82.6	0.035
G3	75.0	66.4	0.720
G4	—	32.4	—
G5	—	100.0	—
G6	—	77.8	—

The results represent “%”.

**Table 3:** Age adjusted and mutually adjusted associations between clustering of four or more HCV risk factors and Aboriginal status, characteristics of HCV infection, and markers of socioeconomic status.

Variable	Age adjusted			Mutually adjusted		
	Odds ratio	95% CI	*p* value	Odds ratio	95% CI	*p* value
Aboriginal	2.19	(1.22; 3.91)	0.008	2.36	(1.22; 4.59)	0.011
Male	2.12	(1.73; 2.60)	0.000	1.99	(1.59; 2.51)	0.000
Genotype 1	1.39	(1.14; 1.70)	0.001	1.36	(1.09; 1.70)	0.007
Transfusions	0.57	(0.46; 0.71)	0.000	0.53	(0.41; 0.69)	0.000
High social deprivation	1.66	(1.37; 2.01)	0.000	1.47	(1.17; 1.86)	0.001
High material deprivation	1.41	(1.13; 1.78)	0.003	1.34	(1.01; 1.77)	0.041
Resident of low income neighborhood	1.31	(1.08; 1.58)	0.006	1.04	(0.79; 1.37)	0.763
Poor housing suitability	1.20	(0.99; 1.46)	0.062	1.09	(0.87; 1.37)	0.463

**Table 4:** Age and sex adjusted and mutually adjusted associations between achieving a sustained virologic response (SVR) and Aboriginal status, characteristics of HCV infection, and markers of socioeconomic status.

Variable	Age and sex adjusted			Mutually adjusted		
	Odds ratio	95% CI	*p* value	Odds ratio	95% CI	*p* value
Aboriginal	0.78	(0.28; 2.16)	0.630	0.79	(0.26; 2.40)	0.678
Genotype						
2	3.49	(1.85; 6.61)	0.000	2.99	(1.56; 5.75)	0.001
3	1.34	(0.88; 2.05)	0.171	1.46	(0.92; 2.30)	0.107
4	0.41	(0.19; 0.87)	0.021	0.34	(0.15; 0.78)	0.011
5	2.28	(0.70; 7.39)	0.169	2.65	(0.69; 10.16)	0.154
4+ HCV risk factors	0.92	(0.67; 1.26)	0.592	0.94	(0.67; 1.34)	0.746
Baseline viral load (>400,000)	0.59	(0.41; 0.86)	0.006	0.56	(0.37; 0.84)	0.006
Advanced fibrosis (F3,4)	0.65	(0.44; 0.96)	0.031	—		
Resident of low income neighborhood	0.64	(0.46; 0.88)	0.006	0.65	(0.44; 0.96)	0.032
High material deprivation	0.63	(0.43; 0.94)	0.022	0.77	(0.49; 1.23)	0.280
High social deprivation	0.65	(0.47; 0.90)	0.010	—		
Poor housing suitability	0.99	(0.72; 1.38)	0.970	—		
